# Mast Cells are Dependent on Glucose Transporter 1 (GLUT1) and GLUT3 for IgE-mediated Activation

**DOI:** 10.1007/s10753-024-02011-8

**Published:** 2024-04-03

**Authors:** Mirjana Grujic, Md Abdul Alim, Lars Hellman, Magnus Peterson, Gunnar Pejler

**Affiliations:** 1 Uppsala University, Department of Medical Biochemistry and Microbiology, Uppsala, Sweden; 2 Uppsala University, Department of Public Health and Caring Sciences, General Medicine, Uppsala, Sweden; 3 University of Cambridge, Division of Immunology, Department of Pathology, Cambridge, UK; 4 Uppsala University, Department of Cell and Molecular Biology, Uppsala, Sweden; 5 Academic Primary Health Care, Region Uppsala, Sweden

**Keywords:** mast cells, glucose, glucose transporters, inflammation, IL-6

## Abstract

Mast cells (MCs) are known to have a pathological impact in a variety of settings, in particular in allergic conditions. There is also limited evidence implicating MCs in diabetes, raising the possibility that MC function may be influenced by alterations in glucose levels. However, it is not known whether MCs are directly affected by elevated glucose concentrations. Moreover, it is not known which glucose transporters that are expressed by MCs, and whether MCs are dependent on glucose transporters for activation. Here we addressed these issues. We show that MCs express high levels of both glucose transporter 1 (GLUT1/Slc2A1) and GLUT3 (Slc2A3). Further, we show that the inhibition of either GLUT1 or GLUT3 dampens both MC degranulation and cytokine induction in response to IgE receptor crosslinking, and that combined GLUT1 and GLUT3 inhibition causes an even more pronounced inhibition of these parameters. In contrast, the inhibition of GLUT1 or GLUT3, or combined GLUT1 and GLUT3 inhibition, had less impact on the ability of the MCs to respond to activation via compound 48/80. Elevated glucose concentrations did not affect MC viability, and had no stimulatory effect on MC responses to either IgE receptor crosslinking or compound 48/80. Altogether, these findings reveal that MCs are strongly dependent on glucose transport via GLUT1 and/or GLUT3 for optimal responses towards IgE-mediated activation, whereas MC functionality is minimally affected by elevated glucose levels. Based on these findings, antagonists of GLUT1 and GLUT3 may be considered for therapeutic intervention in allergic conditions.

**Supplementary Information:**

The online version contains supplementary material available at 10.1007/s10753-024-02011-8.

## Introduction

Mast cells (MCs) are immune cells that are particularly abundant at locations close to the external environment, e.g., skin and mucosal surfaces, but they are also found as resident cells in various internal organs, including the pancreas [1–5]. A typical feature of MCs is their high content of secretory granules, which are filled with numerous types of preformed inflammatory mediators, including biogenic amines (e.g., histamine, serotonin), cytokines, growth factors, proteoglycans, lysosomal enzymes and various proteases, the latter encompassing tryptase, chymase and carboxypeptidase A3 [6, 7]. When MCs are activated, which can occur by IgE receptor crosslinking or in response to numerous other regimes [8], they can undergo degranulation, whereby the contents of the granules are released to the exterior. Moreover, MC activation typically results in de novo production and release of a panel of additional pro-inflammatory mediators, including various lipid mediators (prostaglandins, leukotrienes, platelet-activating factor) and a range of chemokines and cytokines [8]. Altogether, MC activation can thus result in the release of a broad spectrum of pro-inflammatory mediators, together promoting a strong inflammatory reaction.

MCs are mostly well known for their detrimental impact in inflammatory settings. In particular, they are implicated as crucial effector cells in various allergic conditions such as asthma, and also in various types of atopic skin diseases [9–13]. Moreover, MCs have been implicated in numerous types of malignancies, where they are considered to have either beneficial or detrimental activities [14]. In addition, there is some evidence linking MCs to diabetes, as based on both clinical and preclinical observations [2, 4]. Notably, this is in line with the general notion that diabetes is associated with inflammatory events [15]. In rodent models for type 1 diabetes (T1D), partly contradicting findings have been reported, in which MCs were shown to be either protective [16], detrimental [17] or redundant [18]. As for T1D, studies in animal models have provided partly conflicting data with regard to the role of MCs in type 2 diabetes (T2D)-like pathology, with some studies pointing to a detrimental impact of MCs [19], whereas other studies support a protective [20] or redundant role of MCs [21, 22]. In clinical studies, increased infiltration of MCs in pancreatic islets has been observed in patients with T1D [23]. It has also been demonstrated that MC numbers and extent of MC degranulation are increased in the skin of diabetic patients [24], and MCs have been implicated in the regulation of wound healing in diabetes [24].

Importantly, despite the relatively extensive documentation of an association between MCs and diabetic conditions, there is still little insight as to whether MCs respond to elevated glucose. Further, there is little knowledge of how MCs gain access to glucose. Importantly, the expression profile for the various glucose transporters (GLUTs) in MCs has not been studied, and it is not known whether MCs are dependent on specific GLUTs for activation. Here we investigated these issues, and show that MCs are dependent on GLUT1 and GLUT3 for optimal activation in response to IgE receptor crosslinking, but are not activated by elevated glucose.

## Materials and Methods

### Isolation and Culture of Bone Marrow-derived MCs

Femoral and tibial bones from the hind leg of C57BL/6 male mice were used for isolation of bone-marrow cells. The cells were then cultured in Dulbecco's Modified Eagle Medium (DMEM) containing 25 mM glucose and pyruvate, supplemented with 10% heat-inactivated fetal bovine serum, 60 µg/ml penicillin, 50 µg/ml streptomycin sulphate, 2 mM L-glutamine and 20 ng/ml recombinant mouse IL-3. The cells were maintained at 0.5–1 × 10^6^ cells/ml by weekly changes of medium. From day 10, 50% of the cells were cultured in supplemented DMEM media as above but containing only 5 mM glucose. The obtained BMMCs, maintained either in media with 5 mM glucose or 25 mM glucose, were used in experiments.

### Viability of MCs

Cell viability was monitored using the PrestoBlue reagent (C.N. #A13261; Invitrogen, CA) or with trypan blue (#T10282, ThermoFisher Scientific, Watham, MA); cells were mixed with trypan blue at a 1:1 ratio, and cell viability was determined with Countess II FL (ThermoFisher Scientific, Watham, MA).

### Trypsin-like Activity

Ten µl of cell supernatants were mixed with 90 µl H_2_O and 20 µl (1.8 mM) of the chromogenic substrate S-2288 (Instrumentation Laboratory SpA-V, Milano, Italy) in 96-well plates (#82.1581, Sarstedt, Nümbrecht, Germany). Absorbance at 405 nm was monitored.

### Stimulation of Cells

Bone marrow-derived MCs (BMMCs) (1 × 10^6^ cells/ml) were sensitized overnight with IgE anti-DNP (Merck, # D8406-100 µg, Darmstadt, Germany) at 0.1 μg/ml. Next, cells were washed twice with Tyrode's buffer (130 mM NaCl, 5 mM KCl, 1.4 mM CaCl_2_, 1 mM MgCl_2_, 5.6 mM glucose, 10 mM HEPES, and 0.1% BSA, pH 7.4), suspended at 2 × 10^6^ cells/ml either in Tyrode’s buffer (for β-hexosaminidase activity, qPCR analysis) or in cell culture medium (for ELISA) and pipetted into 48-well plates (#83.3923, Sarstedt, Nümbrecht, Germany) at 1 × 10^6^ cells/well. The cells were treated with inhibitors of GLUT1 (Bay876; Merck, # SML1774-5mg, Darmstadt, Germany) or GLUT3 (G3iA; ChemDiv, #D430-1191, San Diego, CA) for 1 h, followed by activation by adding DNP-HSA (0.5 μg/ml) (Merck, #A6661-100mg, Darmstadt, Germany). For β-hexosaminidase activity and mRNA expression, MCs were activated for 1 h. For ELISA measurements, activation with DNP-HSA was performed for 4 h. For stimulation with C48/80 (Merck, #C2313, Darmstadt, Germany), the cells were suspended either in Tyrode’s buffer or in cell culture medium (2 × 10^6^ cells/ml), pipetted into 48-well plates (1 × 10^6^ cells/well), and pretreated with GLUT1 or/and GLUT3 inhibitor. After 1 h, cells were activated with 50 µM C48/80 for 60 min (for β-hexosaminidase activity and qPCR analysis) or 4 h (ELISA).

### Peritoneal Cell-derived MCs (PCMCs)

Abdominal cavities of C57BL/6 male mice were rinsed with 7 ml Tyrode`s buffer (130 mM NaCl, 5 mM KCl, 1.4 mM CaCl_2_, 1 mM MgCl_2_, 5.6 mM glucose, 10 mM HEPES and 0.1% BSA; pH 7.4). Collected cells were rinsed twice with Tyrode’s buffer and once with Dulbecco's Modified Eagle Medium (DMEM) containing glutamax and pyruvate, supplemented with 10% heat-inactivated fetal bovine serum, 60 µg/ml penicillin, 50 µg/ml streptomycin sulphate, 1 mM non-essential amino acids, 50 µM β-mercaptoethanol, 20 ng/ml recombinant mouse IL-3 and 20 ng/ml recombinant mouse stem cell factor (SCF). Peritoneal cells from individual mice were cultured in medium as above at 0.5 × 10^6^ cells/ml. After three days, all cells were pooled. On day 16, the cells were analyzed for the expression of c-kit (anti c-kit-FITC: Invitrogen, ref.# 11-1171-82) and FcɛR1 (anti FcɛR1-PE: eBiosciences, ref.# 12-5898-82) using a BD Accuri C6 Plus flow cytometer (BD Biosciences). Cytospin slides of cells were stained with toluidine blue and pictures were taken with a Nikon 90i microscope (Nikon Instruments Europe, Amsterdam, Netherlands). Mature PCMCs were split into two cultures, one maintained in 5 mM glucose and the other in 25 mM glucose.

### Glucose Treatment

Bone marrow-derived MCs (BMMCs) were cultured in complete DMEM media with 5 mM glucose. When studying the effect of glucose on cell morphology and degranulation of BMMCs, the glucose concentration was adjusted to 12.5 mM, 15 mM and 25 mM with 2 M glucose (VWR, ref.# 101174Y) prepared in H_2_O.

### ELISA

Cytokine levels in media from treated cells was determined by ELISA kits: mouse IL-6 (ThermoFisher Scientific; # 88-7064) and mouse TNFα (Thermo Fischer; #88-7324).

### Cell Morphology and Degranulation

To examine morphological effects of glucose on MCs, cytospin slides were prepared using approximately 0.5 × 10^4^ cells/slide. Cells were stained with toluidine blue. MC degranulation (∼2 × 10^6^ cells in Tyrode’s buffer) was quantified by measuring β-hexosaminidase release [25].

### Immunofluorescence and Confocal Microscopy

For immunofluorescence analysis, cells were first settled onto glass slides and fixed with cold acetone (-20 °C) for 15 min. Staining with antibodies was performed following the protocol described [26]. Briefly, slides were first blocked with normal horse and or goat serum, followed by rinsing with PBS (3×) and incubation overnight (at 4 °C, in dark) with the primary antibody: rabbit monoclonal to GLUT1 (1:100; C.N #ab115730, Abcam, Amsterdam, Netherlands), or rabbit polyclonal to GLUT3 (1:100; C.N #ab234756, Abcam, Amsterdam, Netherlands). All primary antibodies were diluted with PBS/1% BSA/0.3% Triton-X100 to permeabilize the cells. Next, cells were washed in PBS (3 × 5 min), and then incubated for 60 min (on a shaker) at room temperature with biotinylated secondary antibody. As secondary antibody, biotinylated goat anti-rabbit IgG (#BA1000) or biotinylated horse anti-rabbit IgG (#BA1100, Vector Laboratories, Burlingame, CA; 1:250 dilution in PBS/1% BSA) was used. Finally, slides were incubated with streptavidin-Cy2 (for GLUT1; 1:2,000, Amersham Int., Poole, UK) or streptavidin-Cy3 (for GLUT3; 1:5,000, Amersham Int. Poole, UK). For visualization of nuclei, DAPI (4,6-diamidino- 2-phenylindole, Invitrogen, CA) staining was performed (1:500 diluted in PBS). Digital images were captured by a confocal microscope (Zeiss LSM700, Carl Zeiss, Berlin, Germany) and analyzed with ZEN 2009 software (LSM 710; Carl Zeiss, Berlin, Germany). All photographs were taken at original magnifications of 200×, 400× or 630× with oil objective.

### RNA Isolation and qPCR

Total RNA was isolated using the NucleoSpin^®^ RNA isolation kit (MACHEREY-NAGEL, Düren, Germany). RNA purity and concentration was measured by a NanoDrop™ 2000 Spectrophotometer (Thermo Scientific™, Wilmington, DE) and the ND-1000 V3.7.0 program. First-strand cDNA was synthesized using 200 ng of total RNA as template and the iScript cDNA synthesis kit (Bio-Rad, Hercules, CA), on a SimpliAmp Thermal Cycler instrument (Applied Biosystems by Life Technologies/Thermo Fisher Scientific, Darmstadt, Germany). Subsequently, qPCR was performed using up to 100 ng of cDNA, 400 nM primers (indicated below) and iTaq Universal SYBR Green Supermix (Bio-Rad, Hercules, CA), following the PCR cycling conditions recommended by the manufacturer (on a C1000 Touch Thermal Cycler instrument (Bio-Rad, Hercules, CA). Each sample was analyzed in duplicates, and qPCR data was analyzed by using the Bio-Rad CFX Maestro program. The primer efficiency of each primer pair was checked by the iTaqTM Universal SYBR^®^ Green (BioRad, Hercules, CA) Supermix protocol. When a satisfactory primer efficiency (range between 80–110%) and dissociation curve (slope -3.1 to -3.6) was obtained, a qPCR reaction was run in 384-well microtiter plates (Sarstedt, Nümbrecht, Germany) with 5 min centrifugation (2,000 × g) prior to qPCR (CFX384 Touch™, BioRad). The cycling conditions were as follows: Step 1: 95 °C (10 min); step 2: 95 °C (15 s); step 3: 60 °C (60 s); step 4: 72 °C (20 s). Repeat steps 2–4, 40× + dissociation stage (BioRad).

Primer sequences were as follows: GLUT1/Slc2A1, forward: 5′-TCAACACGGCCTTCACTG-3′; GLUT1/Slc2A1, reverse: 5′-CACGATGCTCAGATAGGACATC-3′; GLUT3/Slc2A3, forward: 5′-ATGGGGACAACGAAGGTGAC-3′; GLUT3/Slc2A3, reverse: 5′-CAAAGCTATCACGGAGATGACG-3′; IL-6 forward: 5´-AGACAAAGCCAGAGTCCTTCAGAGA-3´; IL-6, reverse: 5´-TAGCCACTCCTTCTGTGACTCCAGC-3´; TNFα, forward: 5′-CCACATCTCCCAGAAAA-3´; TNFα, reverse: 5′-AGGGTCTGGGCCATAGAACT-3′; Ccl4, forward: 5′- TTCCTGCTGTTTCTCTTACACCT-3´; Ccl4, reverse: 5′- CTGTCTGCCTCTTTTGGTCAG-3′; Gapdh, forward: 5′-CTCCCA CTCTTCCACCTTCG-3´; Gapdh, reverse: 5′-CCACCACCCTGTTGCTGTAG-3′.

### AmpliSeq Transcriptome Analysis

Total RNA was isolated using NucleoSpin^®^ RNA II (Macherey Nagel, 740955) and was used for AmpliSeq transcriptome analysis as described [27]. The displayed data were derived from previous studies [28–30].

### Statistical Analysis

All data were analyzed with one or two-way analysis of variance (Anova) followed by Tukey's or Dunnett`s multiple comparisons test. The results were analyzed using GraphPad Prism 8.1.0 (GraphPad Software, CA). *p*-values ≤ 0.05 were considered to represent statistical significance. The presented data are either the average of two independent experiments or from individual experiments, representative of at least 2 independent experiments.

## Results

### Elevated Glucose Levels are not Toxic to MCs and do not Cause Degranulation of Bone Marrow-derived MCs

**Fig. 1:**
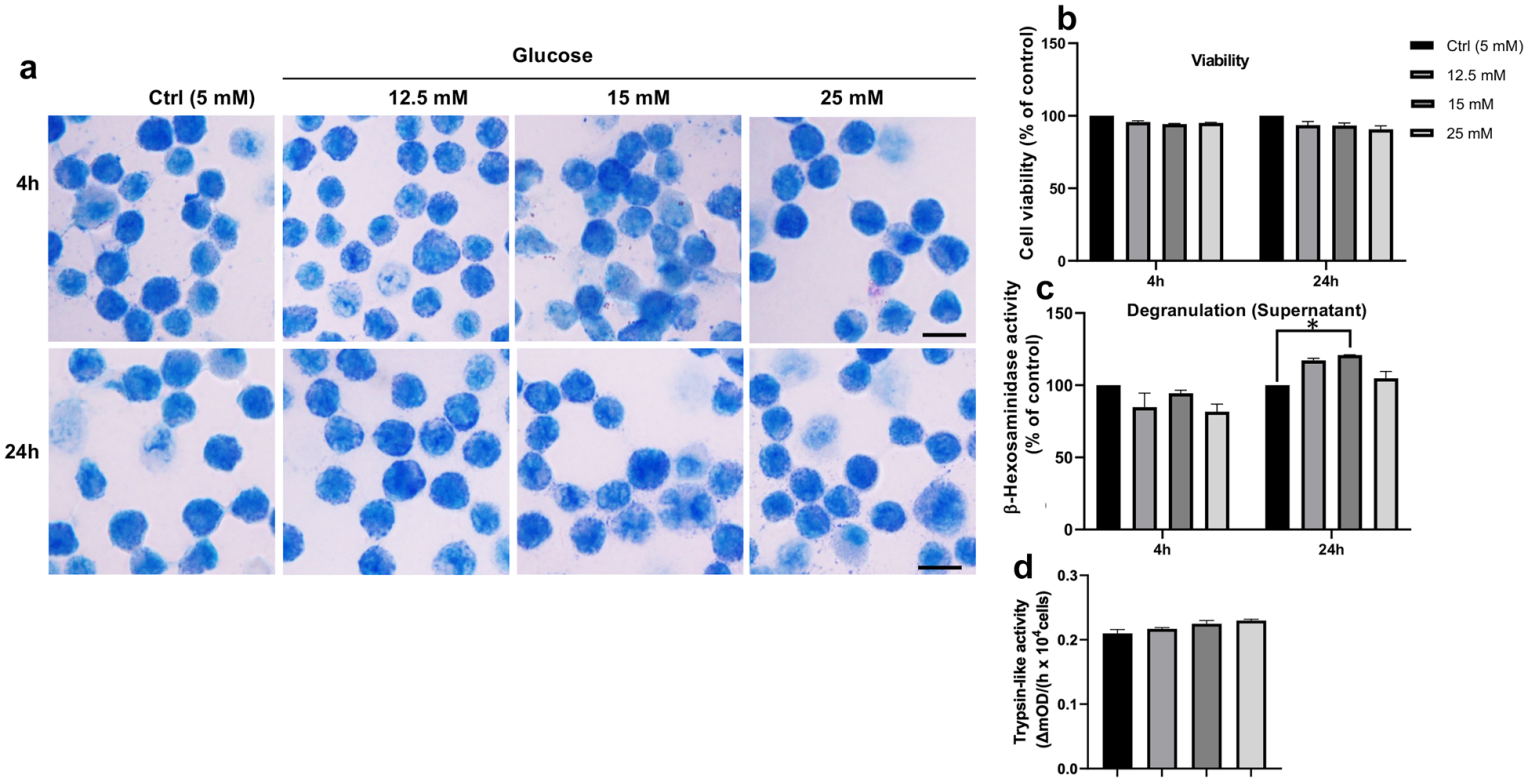
Effect of elevated glucose on MC morphology, degranulation and viability. **a** MCs (BMMCs) were treated with glucose at the indicated concentrations [5 mM (control; Ctrl), 12.5 mM, 15 mM and 25 mM] and time periods (4 h and 24 h), followed by preparation of cytospin slides and staining with toluidine blue. **b** Cell viability of MCs treated with elevated glucose, normalized to controls. **c**,** d** MCs were treated with glucose as indicated, and the amount of released β-hexosaminidase (normalized to controls; **c**) or trypsin-like activity (**d**) was measured. Data in **b**–**d** represent mean values ± SEM (n = 3). Scale bars: 10 μm.

To assess whether MCs are sensitive to elevated glucose levels, we developed primary MCs from mouse bone marrow (bone marrow-derived MCs; BMMCs) and subjected these to either normal (5 mM) or elevated glucose levels (up to 25 mM). First, we assessed whether elevated glucose levels can affect the morphology of the BMMCs. However, as depicted in Fig. 1a, elevated glucose levels (up to 25 mM) did not cause any noticeable morphological effects on the BMMCs, and cell viability was not affected by elevated glucose (Fig. 1b). Further, glucose elevation did not cause excessive degranulation, as assessed by the spontaneous release of the MC granule markers β-hexosaminidase (Fig. 1c) or tryptase (Fig. 1d).

### GLUT1 and GLUT3 are the Major Glucose Transporters Expressed by MCs

**Fig. 2:**
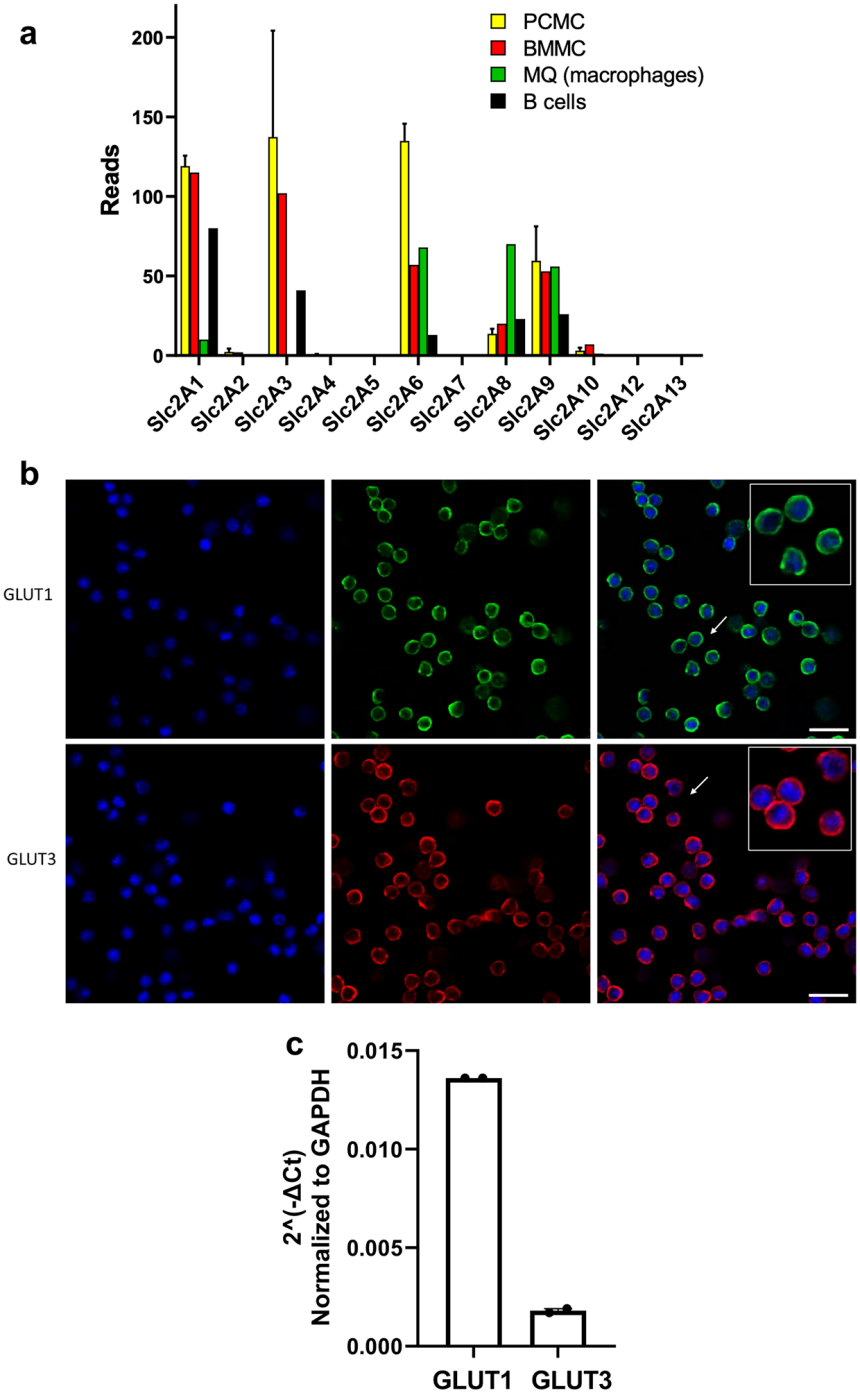
MCs express GLUT1 and GLUT3. **a** Expression of glucose transporters (GLUTs) of the Slc2A family in MCs, as based on AmpliSeq transcriptome analysis. AmpliSeq analysis was performed on peritoneal cell-derived MCs (PCMC; n = 4) and bone marrow-derived MCs (BMMC; single analysis). For comparison, AmpliSeq transcriptome analyses of peritoneal macrophages (MQ) and peritoneal B-cells were included. Data are given as normalized reads. Data for Slc2A11 and Slc2A14 are not included in the AmpliSeq platform. Slc2A1-13 code for GLUT1-GLUT13, respectively. **b** Immunofluorescence analysis confirming the expression of GLUT1 and GLUT3 in MCs, cultured under normal glucose concentrations (5 mM). Size bar, 20 μm. **c** qPCR analysis of the expression of mRNA encoding GLUT1 and GLUT3, presented relative to glyceraldehyde 3-phosphate dehydrogenase (Gapdh) expression. Results are presented as mean values ± SEM (n = 2).

Glucose uptake is mediated by a panel of different glucose transporters, denoted GLUT1 to GLUT14, of which GLUT1-4 are the most well-characterized [31]. These GLUT isoforms have different tissue distribution and different functional characteristics. For example, GLUT4 represents the insulin-dependent glucose transporter, GLUT2 is the liver isoform, GLUT1 is widely expressed among different cellular niches and GLUT3 is generally considered as the main glucose transporter of the brain [31]. To investigate the profile of GLUT expression in MCs, we first assessed global transcriptomic data for expression levels of the various GLUT isoforms (data derived from [28–30]). This analysis revealed that MCs express high levels of GLUT1 (Slc2A1) and GLUT3 (Slc2A3), but very low levels of GLUT2 (Slc2A2) or GLUT4 (Slc2A4) (Fig. 2a). Substantial expression was also seen for GLUT6 (Slc2A6), a lysosomal glucose transporter that does not appear to have a role in glucose uptake into cells [32]. We also noted that the MCs express detectable levels of GLUT8 (Slc2A8), a GLUT isoform with a role in fructose uptake [33]. Further, MCs expressed GLUT9 (Slc2A9), a GLUT isoform which has been shown to mediate urate uptake [34]. The expression of other GLUT isoforms was low to undetectable (Fig. 2a). Similar expression patterns for GLUT isoforms were seen in bone marrow-derived MCs (BMMCs) and in primary MCs developed by expansion of peritoneal cell-derived MCs (PCMCs) (Fig. 2a). As a comparison, peritoneal macrophages expressed considerably lower levels of GLUT1 and GLUT3 in comparison with the MCs, whereas GLUT6 expression was similar in macrophages and MCs (Fig. 2a). Further, peritoneal B-cells showed lower expression levels for GLUT1 and GLUT3 in comparison with MCs, and B-cells expressed lower levels of GLUT6 (Fig. 2a).

The expression of GLUT1 and GLUT3 in MCs was confirmed by immunocytochemical analysis (Fig. 2b) and the expression of mRNA encoding GLUT1 and GLUT3 was additionally confirmed by qPCR analysis (Fig. 2c).

### GLUT1 and GLUT3 are Required for Optimal Degranulation of Bone Marrow-derived MCs in Response to IgE Receptor Crosslinking

**Fig. 3:**
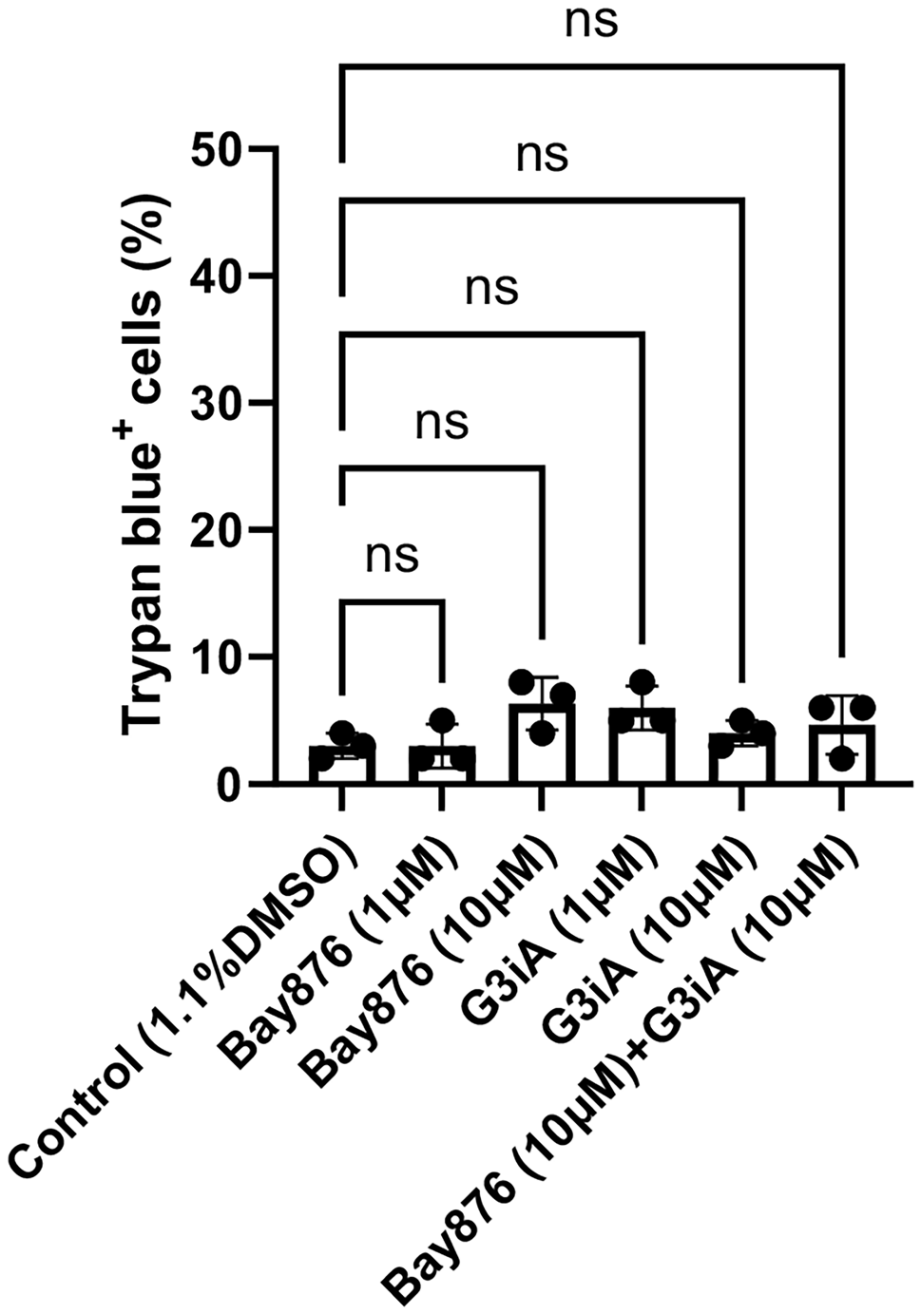
GLUT1/GLUT3 antagonists are not cytotoxic for MCs. MCs (100,000 cells) were treated for 24 h with GLUT1 (Bay876) or GLUT3 (G3iA) antagonists, or by combined treatment with GLUT1 + GLUT3 antagonists. Cells were then mixed with trypan blue and the cell viability was determined. Results are presented as mean values ± SEM (n = 3). Data are from one experiment, representative of two independent experiments. 1-way Anova and Dunnett’s multiple comparison test. ns, not significant.

Having shown that MCs predominantly express GLUT1 and GLUT3 out of the panel of known glucose transporters, we next proceeded to study the role of these GLUTs in MC responses by adopting specific GLUT1 and GLUT3 antagonists. For this purpose, we used the GLUT1 antagonist Bay876 and the GLUT3 antagonist G3iA [35, 36]. First, we assessed whether either of the GLUT1 or GLUT3 antagonists were cytotoxic for BMMCs. As seen in Fig. 3, neither Bay876 nor G3iA showed any significant cytotoxicity for BMMCs at concentrations up to 10 µM. Further, the combination of Bay876 and G3iA (each at 10 µM) showed no significant cytotoxicity for the BMMCs.

**Fig. 4:**
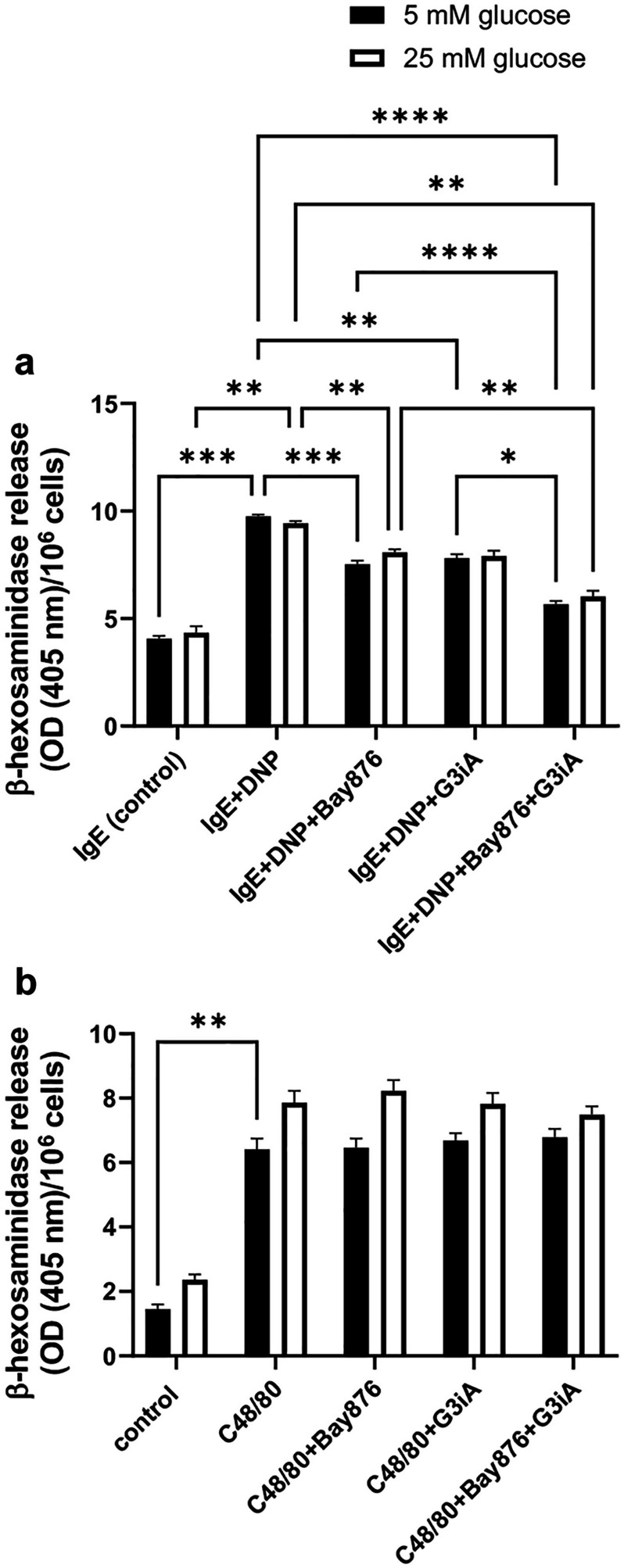
GLUT1 and GLUT3 are required for MC degranulation in response to IgE receptor crosslinking. MCs (BMMCs; 1 × 10^6^ cells) were pretreated for 1 h with either Bay876 (GLUT1 inhibitor; 10 µM) or G3iA (GLUT3 inhibitor; 10 µM), or by the combined treatment with Bay876 + G3iA. For IgE-dependent activation, MCs were first sensitized with IgE anti-DNP prior to the treatment with GLUT inhibitors. Next, MCs were activated by either IgE receptor crosslinking (**a**) or by compound 48/80 (C48/80) (**b**). After 1 h, cell supernatants were collected and analyzed for β-hexosaminidase activity. Cells were cultured either in the presence of normal (5 mM) or elevated (25 mM) glucose levels. Results are presented as mean values ± SEM (n = 4), based on two independent experiments. 2-way Anova and Tukey’s multiple comparison test. *p ≤ 0.05; **p ≤ 0.01; ***p ≤ 0.001; ****p ≤ 0.0001.

By using non-cytotoxic concentrations of these inhibitors (10 µM for both Bay876 and G3iA) we next proceeded to assess the impact of GLUT1/GLUT3 inhibition on MC degranulation. For this purpose, we stimulated the BMMCs by either IgE receptor crosslinking or by exposing the cells to compound 48/80. Compound 48/80 is well-known to induce MC degranulation, and recent data have indicated that compound 48/80 acts through the MRGPRX2 (Mrgprb2 in mice) receptor [37]. MC degranulation was monitored by measuring the release of β-hexosaminidase and, as expected, both IgE receptor crosslinking and stimulation of the MCs by compound 48/80 resulted in significant release of β-hexosaminidase (Fig. 4a, b). Further, it was seen that both the GLUT1 and GLUT3 antagonists had a dampening impact on BMMC degranulation in response to IgE receptor crosslinking, and that combined GLUT1 and GLUT3 antagonism had an even more pronounced inhibitory impact on degranulation. In fact, after combined GLUT1 and GLUT3 inhibition, the amount of β-hexosaminidase release in response to IgE receptor crosslinking was only slightly higher than in baseline controls (Fig. 4a). In contrast, neither the GLUT1 nor the GLUT3 antagonist, or their combination, had any significant impact on degranulation in response to compound 48/80 (Fig. 4b). Further, it was observed that elevated glucose levels (25 mM) had minimal effects on BMMC degranulation in response to either IgE receptor crosslinking or compound 48/80, and elevated glucose levels had no significant impact on the ability of either the GLUT1 or GLUT3 antagonists to inhibit BMMC degranulation (Fig. 4a, b).

### GLUT1 and GLUT3 are Required for Optimal Cytokine Responses in BMMCs Activated by IgE Receptor Crosslinking

**Fig. 5:**
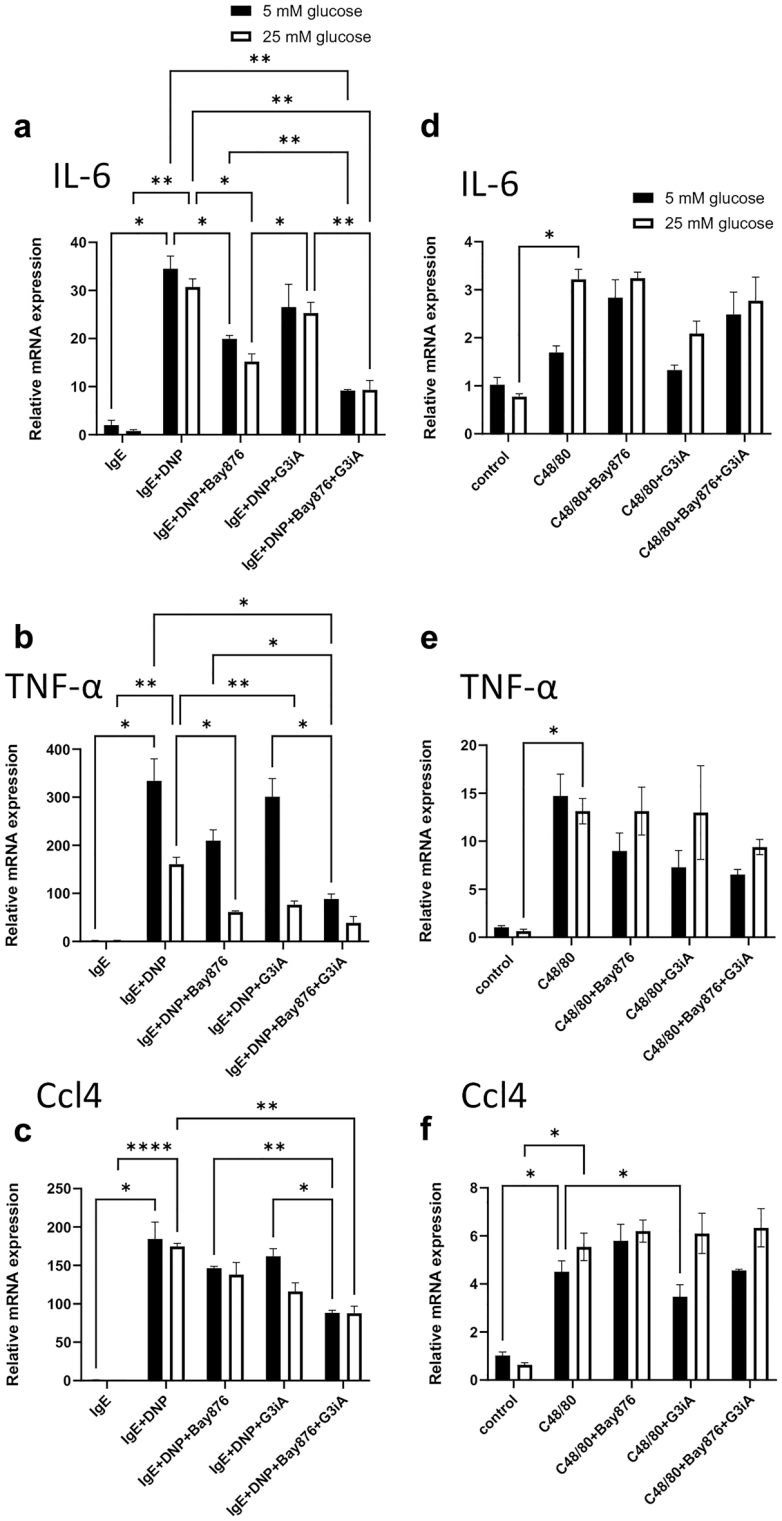
GLUT1 and GLUT3 are required for cytokine gene expression in BMMCs activated by IgE receptor crosslinking. MCs (BMMCs;1 × 10^6^ cells) were pretreated for 1 h with either Bay876 (GLUT1 inhibitor; 10 µM) or G3iA (GLUT3 inhibitor; 10 µM), or by the combined treatment with Bay876 + G3iA. Next, MCs were activated by either IgE receptor crosslinking (**a**–**b**) or by compound 48/80 (C48/80) (**d**–**f**). For IgE-dependent activation, MCs were first sensitized with IgE anti-DNP prior to the treatment with GLUT inhibitors. After 1 h, cells were recovered, followed by total RNA isolation and qPCR analysis. Expression of genes was evaluated relative to glyceraldehyde 3-phosphate dehydrogenase (Gapdh) expression, and normalized to non-activated MCs cultured at normal glucose levels. Results are presented as mean values ± SEM (n = 4) from one individual experiment, representative of 4 independent experiments. 2-way Anova and Tukey’s multiple comparison test. *p ≤ 0.05; **p ≤ 0.01; ***p ≤ 0.001; **** p ≤ 0.0001.

In addition to undergoing degranulation, activated MCs typically respond by de novo synthesis of numerous pro-inflammatory compounds, including various cytokines [8]. Next, we assessed whether such cytokine responses were dependent on GLUT1 and/or GLUT3. As seen in Fig. 5a–c, IgE receptor crosslinking caused an upregulation of IL-6, TNFα and Ccl4 gene expression in BMMCs, and a similar pattern of cytokine upregulation was also seen in response to compound 48/80 (Fig. 5d–f). In the presence of the GLUT1 antagonist, IL-6 gene expression in response to IgE receptor crosslinking was significantly reduced, and there was also a trend of reduction after addition of the GLUT3 antagonist (Fig. 5a). When combining the GLUT1 and GLUT3 antagonists, an even higher extent of IL-6 suppression was seen. Similar patterns were seen for the expression of the TNFα and Ccl4 genes, i.e., combined GLUT1 and GLUT3 inhibition produced a strongly suppressed expression, whereas the single inhibition of either GLUT1 or GLUT3 had less impact (Fig. 5b, c). In contrast to the marked effects on cytokine responses after IgE-dependent MC activation, GLUT1 or GLUT3 blockade, either individually or in combination, had little inhibitory effect on the cytokine gene expression in BMMCs activated by compound 48/80 (Fig. 5d–f).

We also tested whether the expression of the IL-6, TNFα or Ccl4 genes was affected by elevated glucose levels (25 mM). As seen in Fig. 5, the GLUT1 and GLUT3 antagonists effectively inhibited cytokine expression also at elevated glucose concentrations. It was also notable that elevated glucose levels did not cause any increase in the expression of any of the cytokine genes in response to IgE receptor crosslinking (Fig. 5 and Suppl. Fig. 1). In fact, high glucose levels tended to cause a partial suppression of cytokine gene expression, which showed statistical significance for TNFα (Suppl. Fig. 1). In response to compound 48/80, a trend of increased cytokine expression was seen at elevated glucose, but this did not reach statistical significance in most of the cases (see Suppl Fig. 1).

**Fig. 6:**
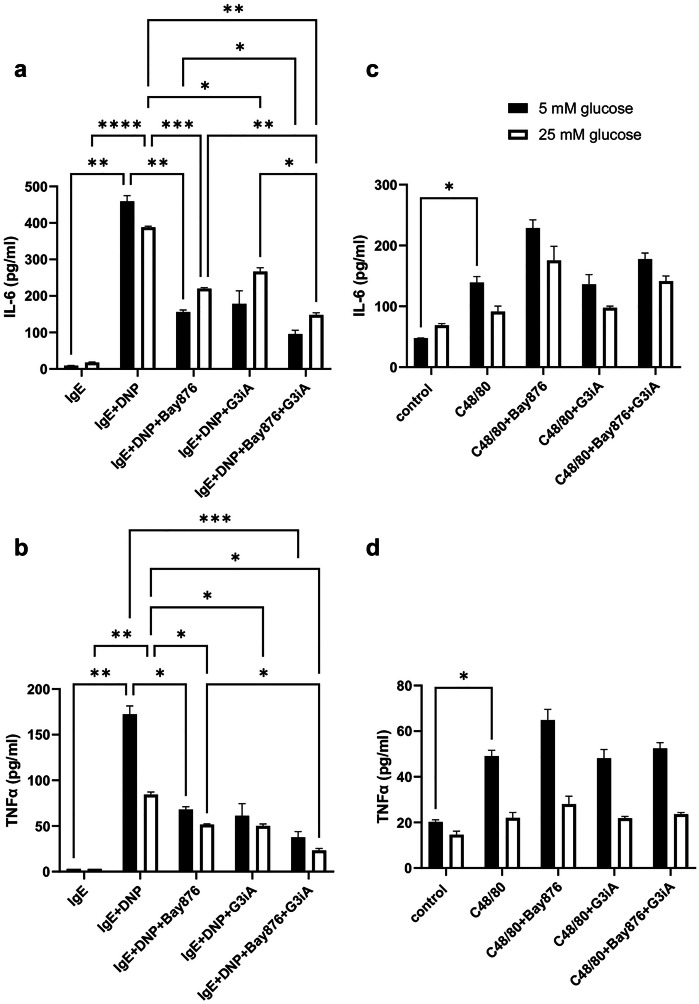
GLUT1 and GLUT3 are required for cytokine production in IgE-activated BMMCs. MCs (BMMCs; 1 × 10^6^ cells) were pretreated for 1 h with either Bay876 (GLUT1 inhibitor; 10 µM) or G3iA (GLUT3 inhibitor; 10 µM), or by the combined treatment with Bay876 + G3iA. Next, MCs were activated by either IgE receptor crosslinking (**a**, **b**) or by compound 48/80 (C48/80) (**c**, **d**). For IgE-dependent activation, MCs were first sensitized with IgE anti-DNP prior to the treatment with GLUT inhibitors. After 4 h, cell supernatants were recovered, followed by measurements of IL-6 and TNFα levels by ELISA. Results are presented as mean values ± SEM (n = 3) from one individual experiment, representative of 2 independent experiments. 2-way Anova and Tukey’s multiple comparison test. *p ≤ 0.05; **p ≤ 0.01.

To verify the effects seen at the mRNA level, we also assessed whether GLUT1 and/or GLUT3 blockade can suppress the release of the corresponding cytokine proteins, as assessed by ELISA measurements. These analyses confirmed the strong induction of both IL-6 and TNFα by IgE receptor crosslinking (Fig. 6a, b). In further agreement with the gene expression analyses, the release of both IL-6 and TNFα in response to IgE receptor crosslinking was reduced by antagonizing either GLUT1 or GLUT3, and combined GLUT1/GLUT3 inhibition caused an even more pronounced reduction in the release of these cytokines (Fig. 6a, b). In further analogy with the mRNA expression data, GLUT1 and/or GLUT3 inhibition effectively inhibited cytokine release also at elevated glucose concentrations (Fig. 6). It was also observed that elevated glucose concentrations did not cause any increase in cytokine output from IgE-activated BMMCs (Suppl. Fig. 2).

Enhanced IL-6 and TNFα protein release was also seen in response to compound 48/80 (Fig. 6c, d). However, the induction of these cytokines by compound 48/80 was less pronounced in comparison with the effects of IgE receptor crosslinking. Similar to the effects at the mRNA level, no significant inhibitory effect of either the GLUT1- or GLUT3 antagonist was seen on the release of IL-6 or TNFα in response to compound 48/80 (Fig. 6c, d). Further, elevated glucose levels had no significant impact on IL-6 release, but partially suppressed TNFα secretion in response to compound 48/80 (Suppl. Fig. 2).

### GLUT1 and GLUT3 are Required for Optimal Degranulation and Cytokine Responses in Activated Peritoneal Cell-derived MCs

**Fig. 7:**
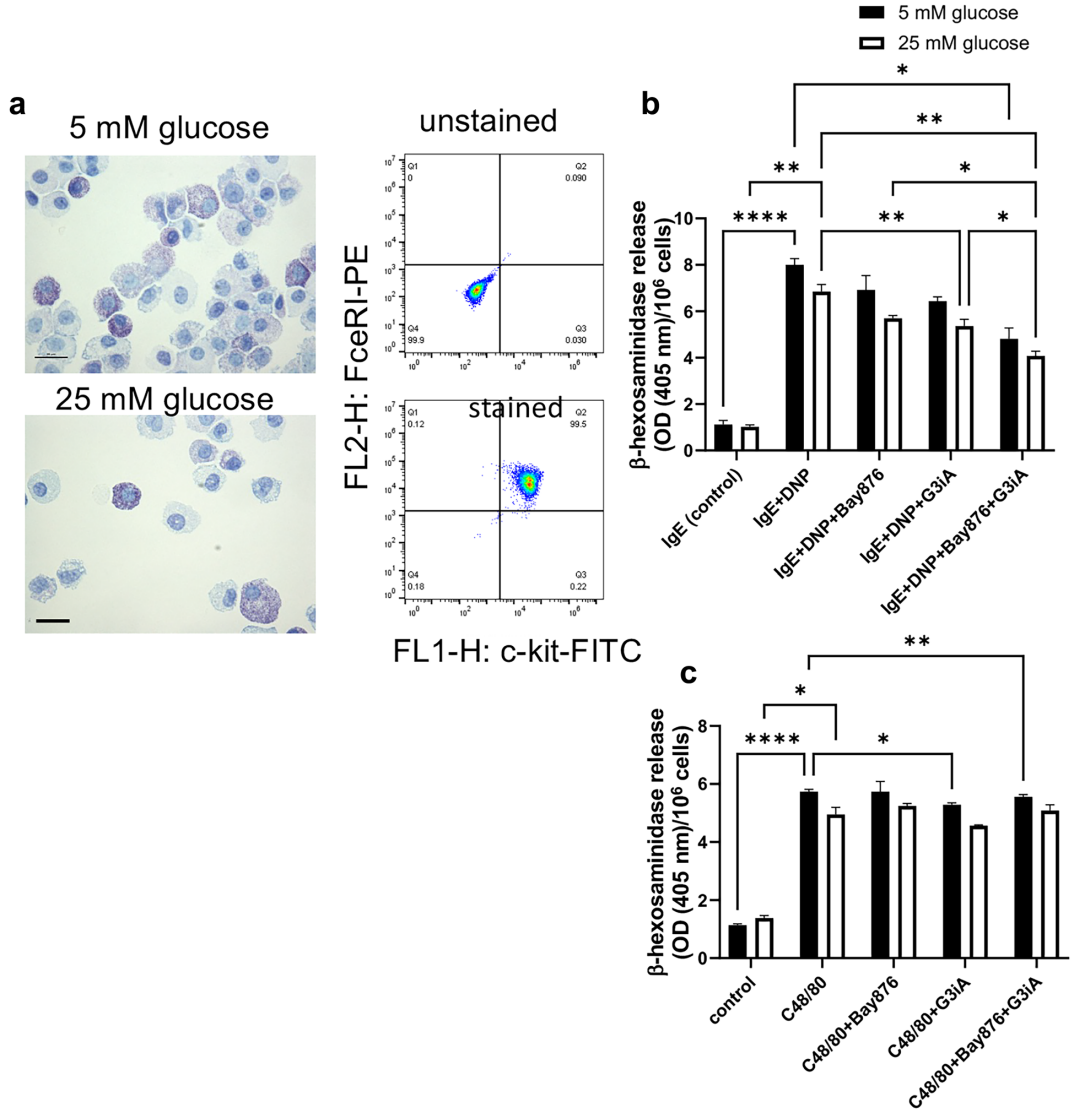
GLUT1 and GLUT3 are required for degranulation of PCMCs in response to IgE receptor crosslinking. **a** PCMCs were developed, cultured in medium containing either 5 or 25 mM glucose, and were stained with either toluidine blue (left panel) or analyzed by flow cytometry for expression of MC markers: FcεRI and c-kit (right panel). **b** PCMCs (1 × 10^6^ cells) were pretreated for 1 h with either Bay876 (GLUT1 inhibitor; 10 µM) or G3iA (GLUT3 inhibitor; 10 µM), or by the combined treatment with Bay876 + G3iA. For IgE-dependent activation, MCs were first sensitized with IgE anti-DNP prior to the treatment with GLUT inhibitors. Next, MCs were activated by either IgE receptor crosslinking (**b**) or by compound 48/80 (C48/80) (**c**). After 1 h, cell supernatants were collected and analyzed for β-hexosaminidase activity. Results are presented as mean values ± SEM (n = 3), taken from one out of two independent experiments. 2-way Anova and Tukey’s multiple comparison test. *p ≤ 0.05; **p ≤ 0.01; ***p ≤ 0.001; ****p ≤ 0.0001.

In the next set of experiments, we asked whether GLUT1 and/or GLUT3 are required for optimal responses in MC populations beyond the bone-marrow derived MCs. For this, we developed MCs from the peritoneal cell population, i.e. peritoneal cell-derived MCs (PCMCs) [38] (Fig. 7a). PCMCs were activated by either IgE receptor crosslinking or by compound 48/80, either in the absence or presence of GLUT1/GLUT3 antagonists. Subsequently, degranulation (β-hexosaminidase release) and cytokine expression were assessed. As seen in Fig. 7b, c  PCMCs underwent degranulation in response to both IgE receptor crosslinking and compound 48/80. Further, it was observed that both the GLUT1 and GLUT3 antagonists alone inhibited IgE-mediated degranulation, and that the combined administration of the GLUT1 and GLUT3 inhibitors caused an even further dampening of the degranulation response. In contrast, GLUT1/GLUT3 inhibition had only a minor inhibitory impact on PCMC degranulation induced by compound 48/80. Experiments were also performed to investigate whether elevated glucose levels impact on the ability of PCMCs to undergo degranulation. As seen in Fig. 7b, c and Suppl. Fig. 3, elevated glucose had only a minimal effect on the degranulation responses, either to IgE receptor crosslinking or to compound 48/80.

**Fig. 8:**
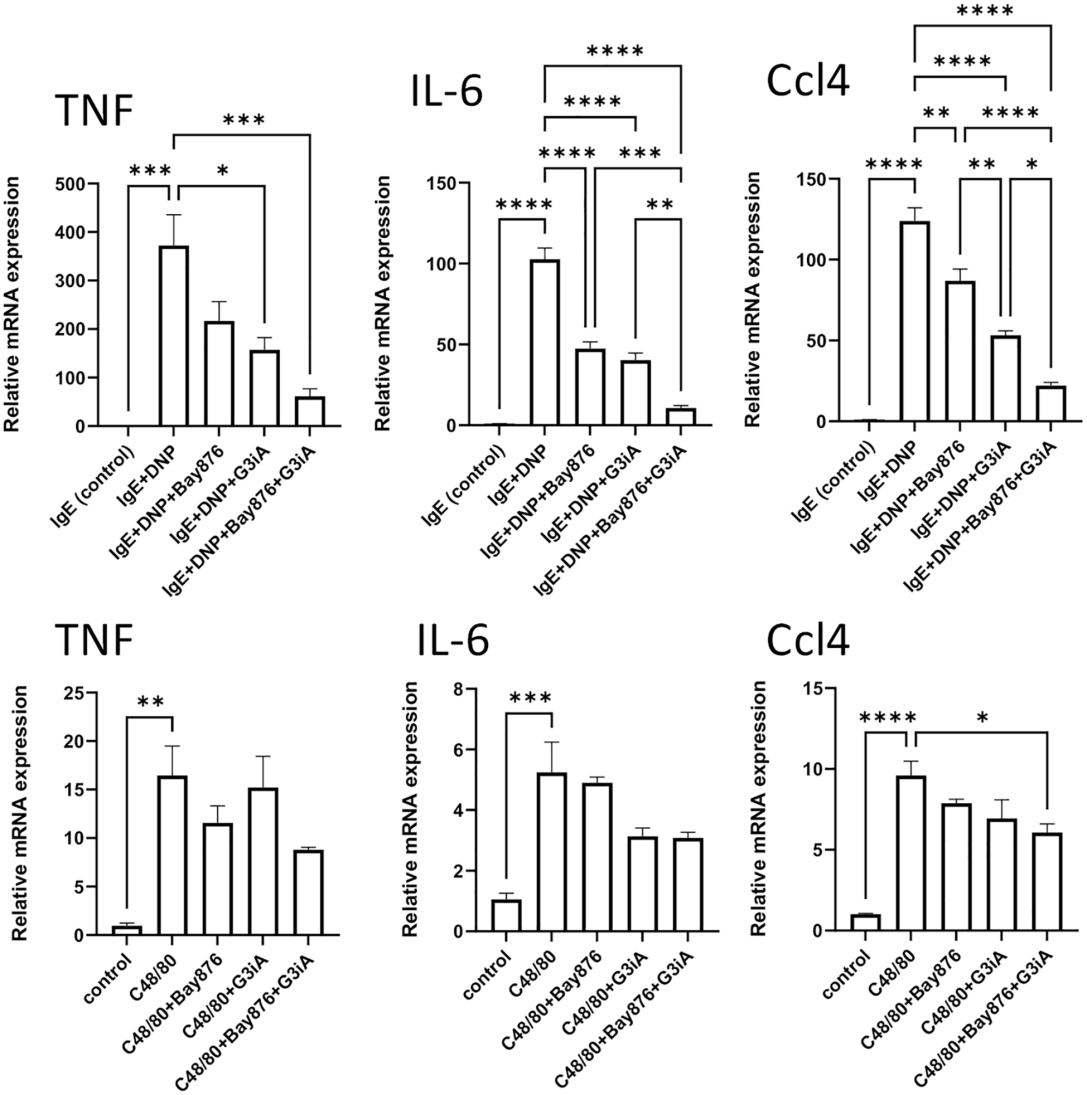
GLUT1 and GLUT3 are required for cytokine gene expression in IgE-activated PCMCs. MCs (PCMCs; 1 × 10^6^ cells) were pretreated for 1 h with either Bay876 (GLUT1 inhibitor; 10 µM) or G3iA (GLUT3 inhibitor; 10 µM), or by the combined treatment with Bay876 + G3iA. Next, MCs were activated by either IgE receptor crosslinking or by compound 48/80 (C48/80). For IgE-dependent activation, MCs were first sensitized with IgE anti-DNP prior to the treatment with GLUT inhibitors. After 1 h, cells were recovered, followed by total RNA isolation and qPCR analysis. Expression of genes was evaluated relative to glyceraldehyde 3-phosphate dehydrogenase (Gapdh) expression, and normalized to non-activated MCs cultured at normal glucose levels. Results are presented as mean values ± SEM (n = 3) from one individual experiment, representative of 2 independent experiments. One-way Anova and Tukey’s multiple comparison test. *p ≤ 0.05; **p ≤ 0.01; ***p ≤ 0.001; ****p ≤ 0.0001.

As for the BMMCs, GLUT1/GLUT3 inhibition caused a profound reduction in the expression of cytokine genes (IL-6, TNF, Ccl4) in response to IgE receptor crosslinking, and combined GLUT1/GLUT3 inhibition caused an even further suppression of the expression of these genes (Fig. 8). In some contrast to the BMMCs, GLUT1/GLUT3 inhibition tended to cause a suppression of cytokine gene expression in response to compound 48/80 (Fig. 8). However, this did not reach statistical significance, except for the effect of combined GLUT1/GLUT3 inhibition on Ccl4 gene expression (Fig. 8).

## Discussion

Based on the relatively extensive documentation implicating MCs in the pathogenesis of diabetes (see "Introduction" section), we here investigated the possibility that MCs may be activated or respond more vigorously to activating stimuli in the presence of elevated glucose levels. However, the results presented here do not support a direct activating impact of glucose elevation on MCs, as assessed by their ability to respond by degranulation or cytokine expression, either at baseline conditions or after challenge with IgE receptor crosslinking or compound 48/80. Hence, these findings do not support the notion that MCs may become activated during diabetic conditions as a result of direct effects of glucose. However, we cannot exclude that, during *in vivo* conditions, indirect effects of elevated glucose concentrations may have an impact on MC function.

Previous findings have revealed that MC activation can have a large impact on cellular metabolism. One important finding is that MC activation by IgE receptor crosslinking leads to a marked increase in glucose utilization through the glycolytic pathway [39]. Intriguingly, although a profound increase in glycolysis was seen in response to IgE stimulation, mitochondrial respiration was not increased to the same extent during acute MC activation [39]. Another interesting observation was that the metabolic response of MCs was dependent on the strength of activation, with high affinity IgE:antigen interaction leading to a strong induction of the glycolytic pathway whereas a weaker antigen:IgE interaction led to a less extensive induction of glycolysis [39]. In agreement with an important role of glucose utilization for MC responses, it has been shown that inhibition of glycolysis by either lactic acid or by other glycolysis antagonists suppresses MC responses to lipopolysaccharide [40]. Moreover, interference with the glycolytic pathway has been shown to inhibit histamine release in activated MCs [41]. Collectively, these findings show that increased glucose utilization is critical for the ability of MCs to respond to activating stimuli.

Despite these notions, it has not previously been known how MCs gain access to glucose following cellular activation, i.e., the contribution of specific glucose transporters to the uptake of glucose into activated MCs has not been revealed. Here we addressed these issues and show that MCs predominantly express GLUT1 and GLUT3 out of the known GLUT family members. Notably, B- and T-lymphocytes have also been shown to express these glucose transporters [42]. We also note that, although lymphocytes and monocytes have been shown to express insulin-dependent glucose transporters (GLUT4) [42–44], MCs appear to lack GLUT4 expression. This suggests that the ability of MCs to utilize glucose is not affected by insulin. We may thus speculate that, under conditions of insulin resistance or low insulin levels, other immune cells such as lymphocytes, monocytes/macrophages and neutrophils may become suppressed as a consequence of their partial dependence on GLUT4 for glucose uptake. In contrast, glucose uptake by MCs would not be restricted under such circumstances.

A major observation in this study was that the ability of MCs to respond to IgE receptor crosslinking is critically dependent on glucose uptake through GLUT1 and GLUT3. This was firstly manifested at the level of MC degranulation, where blockade of either GLUT1 or GLUT3 resulted in a significant inhibition of β-hexosaminidase release. Notably, the combined inhibition of both GLUT1 and GLUT3 led to an even more pronounced inhibition of MC degranulation, suggesting that these two glucose transporters can partly compensate for each other in glucose uptake in response to immunological MC activation. Intriguingly, whereas a profound effect of GLUT1/GLUT3 blockade on IgE-dependent degranulation was seen, no effect of blocking either GLUT1 or GLUT3 was seen on the degranulation of either BMMCs or PCMCs in response to compound 48/80. Compound 48/80 is known to interact with the Mrgprb2 receptor [37], and these findings thus suggest that Mgprb2 ligation, in contrast to IgE-dependent MC activation, does not depend on extensive glucose uptake. In agreement with these findings, it was previously shown that IgE-dependent vs. non-IgE-dependent MC activation lead to differential metabolic responses [45].

Our findings also reveal that the *de novo* cytokine induction in response to IgE receptor crosslinking is strongly dependent on glucose uptake through GLUT1 and GLUT3. As for the MC degranulation response, individual GLUT1 or GLUT3 inhibition caused only a partial blockade of cytokine gene expression, whereas simultaneous GLUT1/GLUT3 blockade had a much more profound impact. Again, these findings suggest that GLUT1 and GLUT3 can partly compensate for each other in mediating the uptake of glucose needed for MCs to respond to activation. Importantly, inhibition of cytokine expression by GLUT1/GLUT3 blockade was seen both at the mRNA and protein level. Similar to the effects on degranulation (β-hexosaminidase release), GLUT1 or GLUT3 blockade did not cause any significant inhibition of the cytokine induction in BMMCs in response to compound 48/80. In contrast, cytokine induction in compound 48/80-stimulated PCMCs appeared to be somewhat susceptible to GLUT1/GLUT3 inhibition. Hence, these findings reinforce the notion that MC activation in response to IgE-dependent vs. non-IgE-dependent regimes can have differential metabolic consequences.

Overall, our findings reveal that MC activation in response to IgE receptor crosslinking is critically dependent on glucose uptake through GLUT1 and GLUT3. Considering that IgE-mediated MC activation is a hallmark event in allergic reactions, our findings raise the possibility that GLUT1/GLUT3 inhibition can have a dampening impact in such settings. Notably, GLUT1 and GLUT3 inhibition is currently emerging as a potential strategy in the treatment of malignant disorders [35, 36], and we may thus envision that the concept of GLUT1/GLUT3 inhibition may potentially be extended to the treatment of allergic disease.

## Supplementary Information

Below is the link to the electronic supplementary material.

Supplementary Fig. 1 Elevated glucose levels have minimal effects on cytokine expression in activated BMMCs. MCs (BMMCs) were cultured at either 5 mM or 25 mM glucose. Cells (1 x 10^6^ cells) were pretreated for 1 h with either Bay876 (GLUT1 inhibitor; 10 µM) or G3iA (GLUT3 inhibitor; 10 µM), or by the combined treatment with Bay876 + G3iA. Next, MCs were activated by either IgE receptor crosslinking (A–C) or by compound 48/80 (C48/80) (D–F). For IgE-dependent activation, MCs were first sensitized with IgE anti-DNP prior to the treatment with GLUT inhibitors. After 1 h, cells were recovered, followed by total RNA isolation and qPCR analysis. Expression of genes was evaluated relative to glyceraldehyde 3-phosphate dehydrogenase (Gapdh) expression, and normalized to non-activated MCs cultured at normal glucose levels. Results are presented as mean values ± SEM (n=4) from one individual experiment, representative of 4 independent experiments. Two-way Anova and Šidák’s multiple comparison test. *p ≤ 0.05; **p ≤ 0.01; ***p ≤ 0.001; **** p ≤ 0.0001. (PDF 89 KB)

Supplementary Fig. 2 Effect of elevated glucose concentrations on the output of cytokines by activated MCs. MCs (BMMCs) were cultured at either 5 mM or 25 mM glucose. MCs (1 x 10^6^ cells) were pretreated for 1 h with either Bay876 (GLUT1 inhibitor; 10 µM) or G3iA (GLUT3 inhibitor; 10 µM), or by the combined treatment with Bay876 + G3iA. Next, MCs were activated by either IgE receptor crosslinking (A, B) or by compound 48/80 (C48/80) (C, D). For IgE-dependent activation, MCs were first sensitized with IgE anti-DNP prior to the treatment with GLUT inhibitors. After 4 h, cell supernatants were recovered, followed by measurements of IL-6 and TNFa levels by ELISA. Results are presented as mean values ± SEM (n=3) from one individual experiment, representative of 2 independent experiments. 2-way Anova and and Šidák’s multiple comparison test. *p ≤ 0.05; **p ≤ 0.01. (PDF 73 KB)

Supplementary Fig. 3 Elevated glucose levels have minimal effects on degranulation in activated PCMCs. MCs (PCMCs) were cultured at either 5 mM or 25 mM glucose. MCs (1 x 10^6^ cells) were pretreated for 1 h with either Bay876 (GLUT1 inhibitor; 10 µM) or G3iA (GLUT3 inhibitor; 10 µM), or by the combined treatment with Bay876 + G3iA. For IgE-dependent activation, MCs were first sensitized with IgE anti-DNP prior to the treatment with GLUT inhibitors. Next, MCs were activated by either IgE receptor crosslinking (A) or by compound 48/80 (C48/80) (B). After 1 h, cell supernatants were collected and analyzed for β-hexosaminidase activity. Results are presented as mean values ± SEM (n=3), based on one out of two independent experiments. 2-way Anova and Šidák’s multiple comparison test. *p ≤ 0.05; **p ≤ 0.01; ***p ≤ 0.001; **** p ≤ 0.0001. (PDF 43 KB)

## Data Availability

All of the data is included in the manuscript.

## Abbreviations

MC: Mast cell
BMMC: Bone marrow-derived mast cell
PCMC: Peritoneal cell-derived mast cell
GLUT: Glucose transporter
T1D: Type 1 diabetes
IL: Interleukin
TNFα: Tumor necrosis factor α
