# Supplementary figures and images for: Mast Cells are Dependent on Glucose Transporter 1 (GLUT1) and GLUT3 for IgE-mediated Activation
Source: Inflammation. 2024 Apr 3;47(5):1820–36. doi: 10.1007/s10753-024-02011-8 (PMC11549158; doi:10.1007/s10753-024-02011-8)

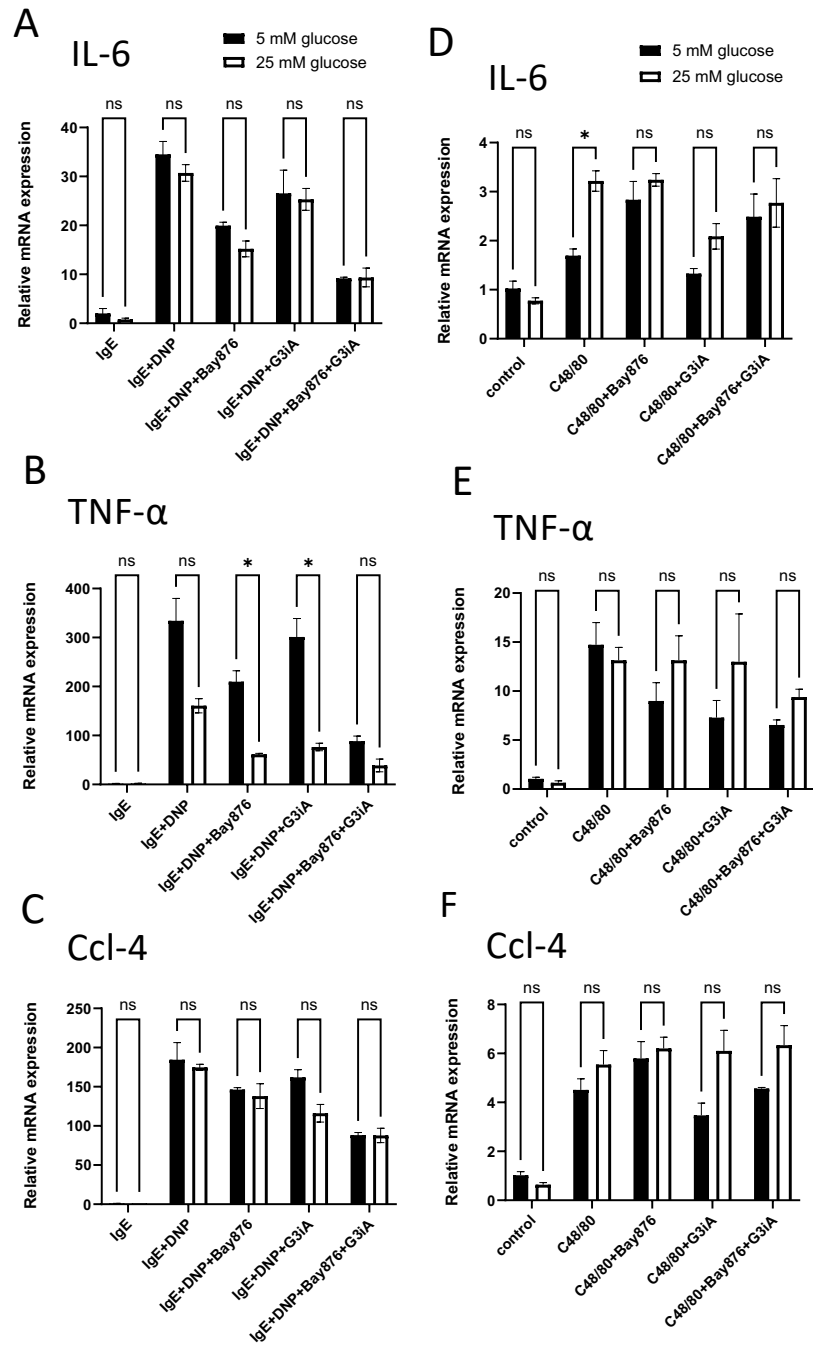

Supplementary Fig 1

Supplement: Supplementary file 1 — Supplementary Fig. 1 Elevated glucose levels have minimal effects on cytokine expression in activated BMMCs. MCs (BMMCs) were cultured at either 5 mM or 25 mM glucose. Cells (1 x 10^6^ cells) were pretreated for 1 h with either Bay876 (GLUT1 inhibitor; 10 µM) or G3iA (GLUT3 inhibitor; 10 µM), or by the combined treatment with Bay876 + G3iA. Next, MCs were activated by either IgE receptor crosslinking (A–C) or by compound 48/80 (C48/80) (D–F). For IgE-dependent activation, MCs were first sensitized with IgE anti-DNP prior to the treatment with GLUT inhibitors. After 1 h, cells were recovered, followed by total RNA isolation and qPCR analysis. Expression of genes was evaluated relative to glyceraldehyde 3-phosphate dehydrogenase (Gapdh) expression, and normalized to non-activated MCs cultured at normal glucose levels. Results are presented as mean values ± SEM (n=4) from one individual experiment, representative of 4 independent experiments. Two-way Anova and Šidák’s multiple comparison test. *p ≤ 0.05; **p ≤ 0.01; ***p ≤ 0.001; **** p ≤ 0.0001. (PDF 89 KB) [file 10753_2024_2011_MOESM1_ESM.pdf]

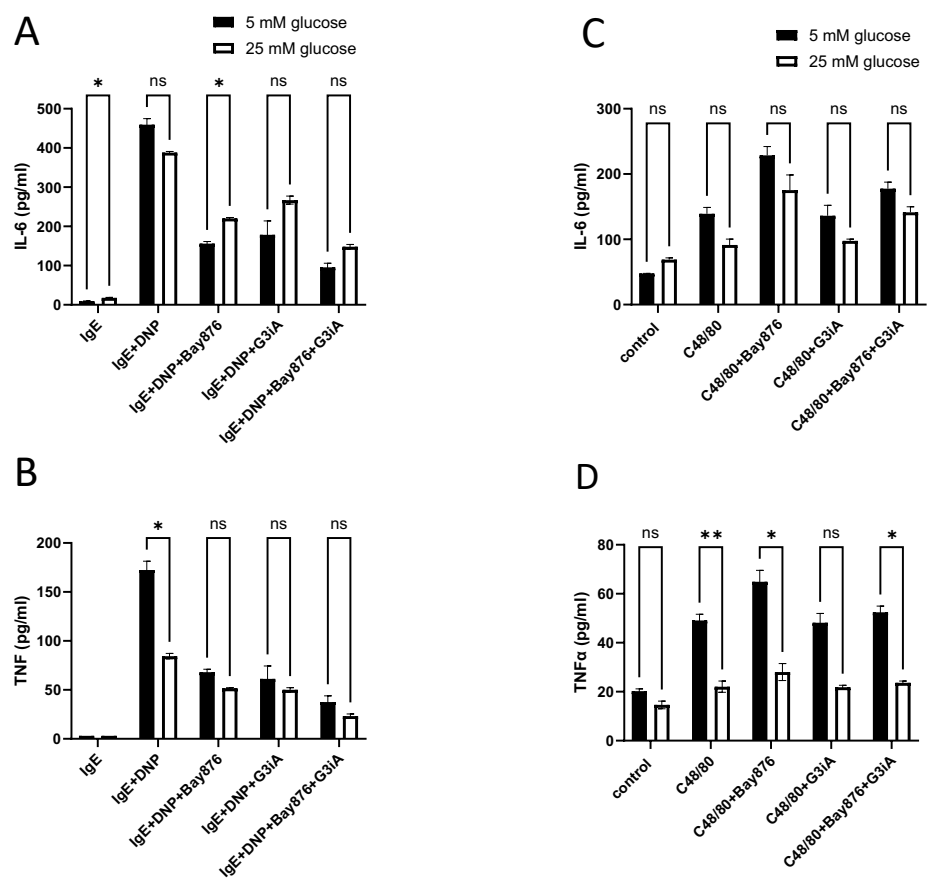

Supplementary Figure 2

Supplement: Supplementary file 2 — Supplementary Fig. 2 Effect of elevated glucose concentrations on the output of cytokines by activated MCs. MCs (BMMCs) were cultured at either 5 mM or 25 mM glucose. MCs (1 x 10^6^ cells) were pretreated for 1 h with either Bay876 (GLUT1 inhibitor; 10 µM) or G3iA (GLUT3 inhibitor; 10 µM), or by the combined treatment with Bay876 + G3iA. Next, MCs were activated by either IgE receptor crosslinking (A, B) or by compound 48/80 (C48/80) (C, D). For IgE-dependent activation, MCs were first sensitized with IgE anti-DNP prior to the treatment with GLUT inhibitors. After 4 h, cell supernatants were recovered, followed by measurements of IL-6 and TNFa levels by ELISA. Results are presented as mean values ± SEM (n=3) from one individual experiment, representative of 2 independent experiments. 2-way Anova and and Šidák’s multiple comparison test. *p ≤ 0.05; **p ≤ 0.01. (PDF 73 KB) [file 10753_2024_2011_MOESM2_ESM.pdf]

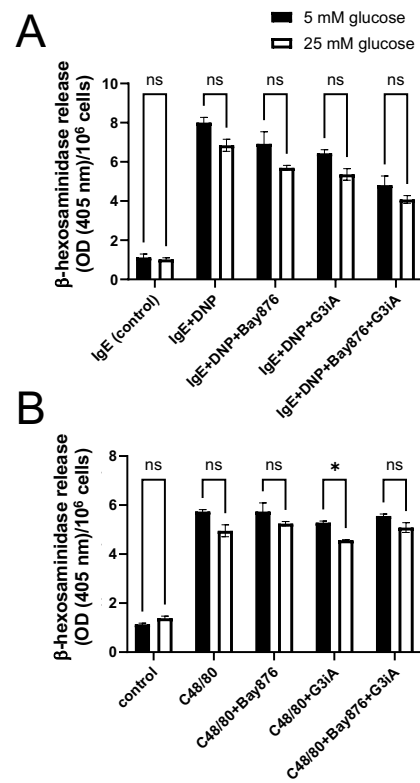

Supplementary Fig. 3

Supplement: Supplementary file 3 — Supplementary Fig. 3 Elevated glucose levels have minimal effects on degranulation in activated PCMCs. MCs (PCMCs) were cultured at either 5 mM or 25 mM glucose. MCs (1 x 10^6^ cells) were pretreated for 1 h with either Bay876 (GLUT1 inhibitor; 10 µM) or G3iA (GLUT3 inhibitor; 10 µM), or by the combined treatment with Bay876 + G3iA. For IgE-dependent activation, MCs were first sensitized with IgE anti-DNP prior to the treatment with GLUT inhibitors. Next, MCs were activated by either IgE receptor crosslinking (A) or by compound 48/80 (C48/80) (B). After 1 h, cell supernatants were collected and analyzed for β-hexosaminidase activity. Results are presented as mean values ± SEM (n=3), based on one out of two independent experiments. 2-way Anova and Šidák’s multiple comparison test. *p ≤ 0.05; **p ≤ 0.01; ***p ≤ 0.001; **** p ≤ 0.0001. (PDF 43 KB) [file 10753_2024_2011_MOESM3_ESM.pdf]
